# Nodal stage is the key prognostic factor in synchronous and metachronous multiple primary colorectal adenocarcinoma after curative-intent resection: a retrospective study

**DOI:** 10.1186/s12957-026-04383-7

**Published:** 2026-04-28

**Authors:** Yuncan Xing, Zeyu Wu, Shiwen Mei, Zheng Wang, Qingshan Wang, Qian Liu

**Affiliations:** Department of Colorectal Surgery, National Cancer Center/National Clinical Research Center for Cancer/Cancer Hospital, Chinese Academy of Medical Sciences and Peking Union Medical College, Beijing, China

**Keywords:** Multiple primary colorectal cancer, Prognosis, Lymph node stage

## Abstract

**Background:**

Multiple primary colorectal cancer (MPCRC) is uncommon but clinically challenging, and differences between synchronous MPCRC (SMPCRC) and metachronous MPCRC (MMPCRC) remain incompletely defined. We compared clinicopathological and surgical features between SMPCRC and MMPCRC and explored prognostic factors for overall survival (OS) in MPCRC.

**Methods:**

This retrospective cohort study consecutively included patients with pathologically confirmed multiple primary colorectal adenocarcinoma who underwent curative-intent resection at our hospital. SMPCRC was defined as tumors identified within 6 months and MMPCRC as a subsequent primary diagnosed after 6 months. Clinicopathological and perioperative variables were extracted from medical records and pathology reports. Mismatch repair protein expression was assessed as an exploratory pathological variable. OS was analyzed using Kaplan–Meier methods and Cox proportional hazards regression.

**Results:**

A total of 165 patients were included (120 SMPCRC and 45 MMPCRC) with follow-up until December 2024. Baseline characteristics were broadly comparable between groups. SMPCRC more frequently underwent laparoscopic surgery (97.5% vs 88.9%, *p* = 0.035) and had a higher lymph node yield (27.86 ± 13.32 vs 20.87 ± 14.07, *p* = 0.004). Vascular invasion was more common in SMPCRC (45.8% vs 26.7%, *p* = 0.026), and N-stage distribution differed between subtypes (*p* < 0.001). OS did not differ significantly between SMPCRC and MMPCRC. In multivariable analysis, N stage remained the only independent predictor of OS (HR 2.979, 95% CI 1.404–6.320, *p* = 0.004).

**Conclusion:**

Although SMPCRC and MMPCRC differed in several perioperative and pathological features, OS was primarily determined by N stage. N stage should be considered the key variable for prognostic stratification in resected MPCRC.

**Supplementary Information:**

The online version contains supplementary material available at 10.1186/s12957-026-04383-7.

## Introduction

Colorectal cancer (CRC) has emerged as the third most prevalent malignancy globally in recent years, but ranks second in terms of mortality [1]. In this context, multiple primary colorectal cancer (MPCRC) has gained increasing clinical attention. MPCRC refers to the synchronous or successive occurrence of two or more independent primary colorectal tumors in the same individual. Based on the temporal interval of diagnosis, MPCRC can be classified into synchronous MPCRC (SMPCRC), in which tumors are identified within six months, and metachronous MPCRC (MMPCRC), in which a subsequent primary tumor is diagnosed more than six months after the first lesion. MPCRC is reported to account for approximately 2% to 10% of CRC cases, and its recognition has increased with improvements in diagnostic evaluation and surveillance [2, 3]. While the pathogenesis of MPCRC is not yet fully elucidated, it is considered multifactorial, with reported associations including lifestyle choices such as smoking, premalignant conditions like ulcerative colitis, and well-defined hereditary syndromes including familial adenomatous polyposis (FAP) and Lynch syndrome (LS) [4–6].

A growing body of evidence suggests that MPCRC differs from unifocal CRC in several clinicopathological and biological characteristics [7–9]. Previous studies have reported that MPCRC, particularly SMPCRC, may be associated with less favorable outcomes, a higher prevalence of aggressive histological features, and enrichment of deficient mismatch repair (dMMR) and related microsatellite instability (MSI) [10]. However, compared with the relatively well-described differences between MPCRC and unifocal CRC, direct comparative evidence between SMPCRC and MMPCRC remains limited [11]. Moreover, a clear consensus regarding the clinicopathological spectrum, prognostic factors, and optimal management strategies for MPCRC remains elusive, largely because published studies are often constrained by small sample sizes, retrospective designs, and variable inclusion criteria. In particular, data focused on patients with multiple primary colorectal adenocarcinoma undergoing curative-intent resection and evaluating survival determinants across the MPCRC population remain limited [12].

Therefore, our study aimed to compare the clinicopathological characteristics and surgical features of patients with multiple primary colorectal adenocarcinoma in a consecutive cohort from a high-volume center, and to identify independent factors associated with survival across the resected MPCRC population, thereby providing evidence to support risk stratification and clinical decision-making in this distinct patient group.

## Methods

### Study design and patient selection

We conducted a retrospective cohort study in the Department of Colorectal Surgery, National Cancer Center, Cancer Hospital, Chinese Academy of Medical Sciences and Peking Union Medical College, China. Patients who underwent curative-intent resection for CRC between January 2012 and December 2022 were screened through the institutional database. Patients meeting the preliminary diagnostic criteria for MPCRC were consecutively identified. The follow-up was updated through December 2024, and survival status was obtained from outpatient documentation and telephone contact when needed. The maximum follow-up duration was 144 months. Patients were considered eligible if more than one primary CRC was present, with at least two anatomically separate lesions and postoperative pathological confirmation of malignancy for each lesion. The determination that lesions represented distinct primary tumors was based on concordant findings from preoperative colonoscopy, radiologic evaluation, operative findings, and pathology review. Patients were also required to have no evidence of a primary malignancy arising outside the colorectum. For metachronous cases, prior CRC resection was required, and the subsequently detected lesion had to be judged as a new primary tumor rather than recurrence at a previous anastomotic site.

**Fig. 1:**
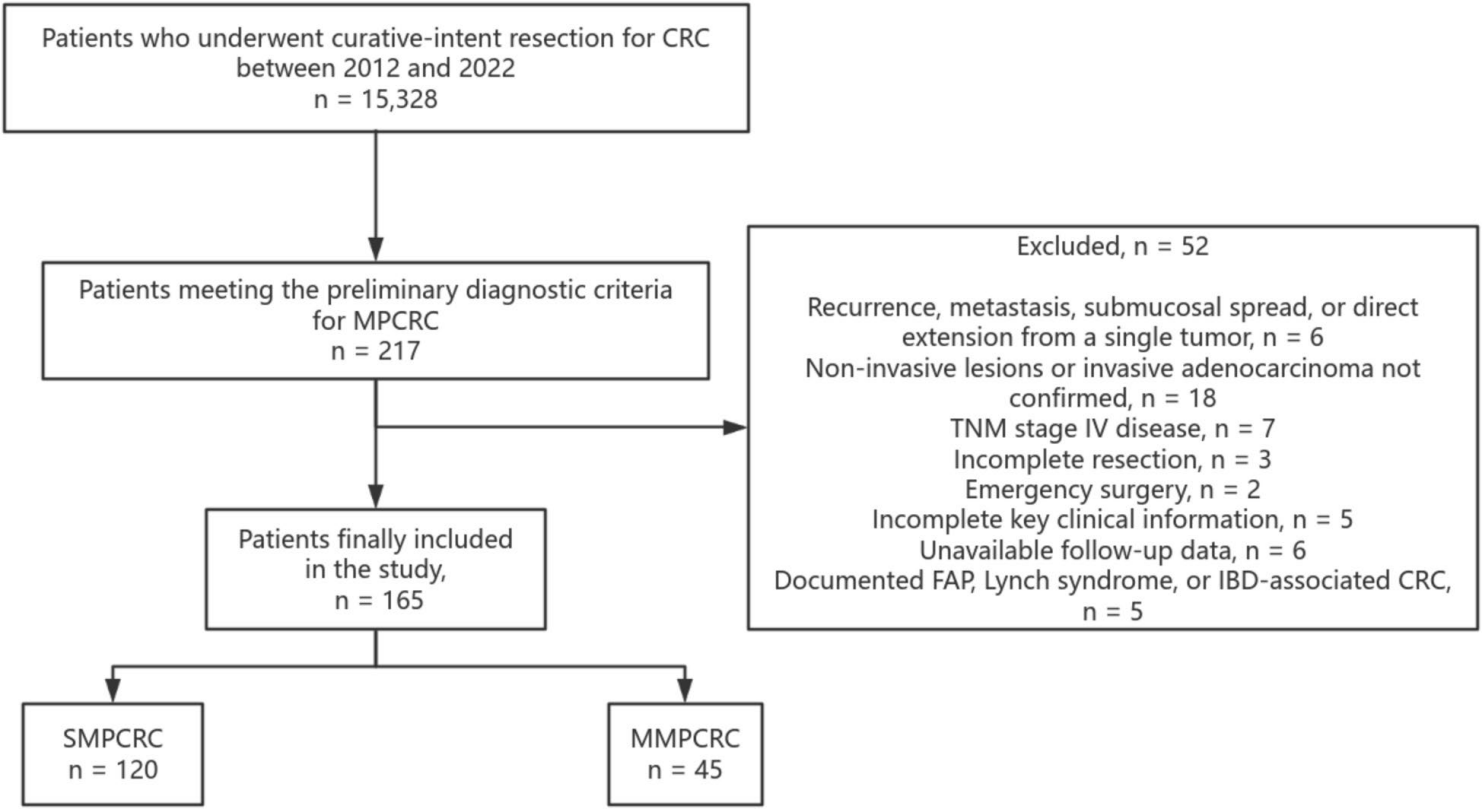
Study flowchart of patient selection. MPCRC, multiple primary colorectal cancer; SMPCRC, synchronous multiple primary colorectal cancer; MMPCRC, metachronous multiple primary colorectal cancer; CRC, colorectal cancer; FAP, familial adenomatous polyposis; LS, Lynch syndrome; IBD, inflammatory bowel disease

Patients were excluded if multiple lesions could be explained by recurrence, metastatic disease, submucosal spread, or direct extension from a single tumor; if non-invasive lesions were present or invasive adenocarcinoma was not confirmed; if TNM stage IV disease was present; or if complete resection was not achieved. Lesions showing only high-grade dysplasia or intraepithelial neoplasia without invasive adenocarcinoma were not considered eligible MPCRC lesions and were not counted toward study inclusion. Patients undergoing emergency surgery, those with incomplete key clinical information, and those lacking follow-up data were also excluded. In addition, patients with a documented clinical diagnosis or prior medical history of FAP, LS, or inflammatory bowel disease-associated CRC were excluded. The patient selection process is summarized in Fig. 1.

### Definitions and classification

MPCRC was defined as two or more distinct primary colorectal carcinomas occurring in the same individual. Synchronous disease referred to tumors detected at initial presentation or within 6 months, whereas metachronous disease referred to a new primary tumor diagnosed more than 6 months after the first CRC.

### Data collection and clinicopathological variables

Baseline information was extracted from electronic medical records, including age, sex, body mass index (BMI), smoking and drinking history, history of abdominal surgery, family history of malignancy, and American Society of Anesthesiologists (ASA) classification. Family history of malignancy was recorded as a general clinical variable and did not imply a diagnosis of hereditary CRC syndrome. Preoperative laboratory results included hemoglobin (Hb) and albumin (ALB), and serum tumor markers included carcinoembryonic antigen (CEA) and carbohydrate antigen 19–9 (CA19-9), which were categorized as normal or elevated according to the institutional upper limits. Operative variables included surgical approach and the number of harvested lymph nodes. Pathological variables were retrieved from postoperative pathology reports and included tumor location, maximum diameter, T stage, N stage, overall pathological stage, differentiation, vascular invasion, perineural invasion, and circumferential resection margin status. Tumor staging followed the American Joint Committee on Cancer (AJCC) Tumor–Node–Metastasis (TNM) staging system, 8th edition.

For patients with multiple tumors, analyses were based on a prespecified representative lesion at the patient level. The representative lesion was defined as the tumor with the highest TNM stage; when tumors shared the same TNM stage, the lesion with the larger diameter was selected. This approach was adopted because survival modeling required one set of tumor-level pathological covariates per patient. We selected the lesion with the highest TNM stage to reflect the tumor with the greatest expected prognostic relevance.

### Assessment of mismatch repair protein expression

Mismatch repair protein expression status was obtained from postoperative pathology reports based on immunohistochemistry (IHC) for MLH1, MSH2, MSH6, and PMS2. Deficient mismatch repair was defined as loss of expression of at least one of these proteins, while proficient mismatch repair required preserved expression of all four proteins. dMMR detected by postoperative immunohistochemistry was not considered equivalent to LS, because abnormal tumor MMR protein expression alone is insufficient to establish a hereditary syndrome diagnosis without further germline testing and clinical genetic evaluation.

### Outcome

Overall survival (OS) was defined at the patient level as the time from curative-intent surgery for the index lesion used for patient-level analysis to death from any cause or last follow-up. For patients with MMPCRC, the index date was the date of curative surgery for the metachronous primary tumor.

### Statistical analysis

Continuous variables are summarized as mean with standard deviation (SD) and were compared using the independent samples *t* test or the Mann–Whitney *U* test according to data distribution. Categorical variables are reported as counts with percentages and were compared using the *chi-*square test or Fisher exact test when appropriate. OS was analyzed using the Kaplan–Meier method with group comparisons by the log-rank test. Prognostic factors were explored using Cox proportional hazards regression. Variables with a univariate *p* value below 0.10 or judged clinically relevant were entered into the multivariable Cox model. Hazard ratios (HR) are reported with 95 percent confidence intervals (CIs), and statistical significance was set at a two-sided *p* value below 0.05. These statistical analyses were performed using Statistical Package for the Social Sciences 26.0 for Windows (SPSS Inc, Chicago, IL) and R software (version 3.5.3).

### Ethics approval and consent to participate

This study was reviewed and approved by the Institute Research Medical Ethics Committee of National Cancer Center. The study was conducted in accordance with the Declaration of Helsinki. Given the retrospective nature of the study and the use of de-identified data, the requirement for informed consent to participate was waived by the Ethics Committee.

## Results

### Baseline characteristics of patients with MPCRC

According to the criteria, the final cohort comprised 165 patients, including 120 SMPCRC and 45 MMPCRC. The baseline characteristics of the 165 patients with MPCRC were presented in Table 1. Overall, demographic and preoperative characteristics were comparable between groups. The proportion of male patients was numerically higher in the SMPCRC group than in the MMPCRC group (72.5% vs. 62.2%), but the difference was not statistically significant (*p* = 0.254). Mean age was similar (63.61 ± 9.26 vs. 63.29 ± 11.99 years, *p* = 0.872), as was BMI (24.09 ± 2.85 vs. 23.69 ± 3.37 kg/m^2^, *p* = 0.444). Most patients in both groups had an ASA class of 1–2 (89.2% vs. 88.9%, *p* = 0.959).

**Table 1:** Baseline characteristics of patients with MPCRC

Baseline information	SMPCRC (*n* = 120)	MMPCRC(*n* = 45)	*p* value
Sex			0.254
Male	87 (72.5%)	28 (62.2%)	
Female	33 (27.5%)	17 (37.8%)	
Age, mean (SD), year	63.61 (9.26)	63.29 (11.99)	0.872
BMI, mean (SD), kg/m^2^	24.09 (2.85)	23.69 (3.37)	0.444
Smoking history	47 (39.2%)	17 (37.8%)	0.870
Drinking history	37 (30.8%)	12 (26.7%)	0.659
Family history of malignancy	31 (25.8%)	10 (22.2%)	0.633
Previous abdominal surgery	26 (21.7%)	9 (20.0%)	0.816
ASA classification			0.959
1–2	107 (89.2%)	40 (88.9%)	
3–4	13 (10.8%)	5 (11.1%)	
Abnormal preoperative CA19-9	63 (52.5%)	22 (48.9%)	0.679
Abnormal preoperative CEA	74 (61.7%)	27 (60.0%)	0.845
Preoperative Hb, mean (SD), g/l	120.3 (19.84)	124.5 (21.32)	0.242
Preoperative ALB, mean (SD), g/l	35.06 (5.06)	36.09 (4.80)	0.239
Presence of polyp	64 (53.3%)	19 (42.2%)	0.204

Values are presented as counts (percentages), unless stated otherwise

*ALB* Albumin, *ASA* American Society of Anesthesiologists, *BMI* Body mass index, *CA19-9* Carbohydrate antigen 19–9, *CEA* Carcinoembryonic antigen, *Hb* Hemoglobin, *MMPCRC* Metachronous multiple primary colorectal cancer, *MPCRC* Multiple primary colorectal cancer, *SD* Standard deviation, *SMPCRC* Synchronous multiple primary colorectal cancer

Regarding medical and personal history, smoking, alcohol use, prior abdominal surgery, and family history of malignancy were numerically more frequent in the SMPCRC group; however, none of these differences reached statistical significance (*p* = 0.870, 0.659, 0.816, and 0.633, respectively). Preoperatively, the proportions of abnormal CA19-9 (52.5% vs. 48.9%, *p* = 0.679) and abnormal CEA (61.7% vs. 60.0%, *p* = 0.845) did not differ significantly between the two groups. Similarly, no significant differences were observed in preoperative hemoglobin levels (120.3 ± 19.84 g/L in SMPCRC vs. 124.5 ± 21.32 g/L in MMPCRC, *p* = 0.242) or albumin levels (35.06 ± 5.06 g/L vs. 36.09 ± 4.80 g/L, *p* = 0.239). The presence of polyps did not differ significantly between the SMPCRC and MMPCRC groups (53.3% vs. 42.2%, *p* = 0.204).

### Clinical, surgical, and pathological characteristics of patients with MPCRC

Table 2 summarizes the clinical, surgical, and pathological characteristics of patients with MPCRC. The most common tumor distribution pattern involved both the left and right colon (52.5% in SMPCRC vs. 57.8% in MMPCRC), followed by tumors located only in the left colon (35.0% vs. 33.3%) or only in the right colon (12.5% vs. 8.9%), with no significant difference in tumor location between the two groups (*p* = 0.754). Laparoscopic surgery was more frequently performed in the SMPCRC group (97.5% vs. 88.9%, *p* = 0.035). Regarding T stage, most primary tumors in the SMPCRC group were classified as T3–T4 (82.5%), compared with 71.1% in the MMPCRC group, though the difference was not statistically significant (*p* = 0.107). For N stage, N2 was the most frequent stage in MMPCRC (71.1%), while SMPCRC cases were predominantly N0 or N1 (40.0% each). The distribution of N stage differed significantly between the two groups (*p* < 0.001). The proportion of stage I–II disease was numerically higher in SMPCRC than in MMPCRC (40.0% vs. 28.9%), but the difference was not statistically significant (*p* = 0.188). The mean tumor size of the SMPCRC was numerically larger than that in the MMPCRC group (4.29 ± 1.51 cm vs. 3.81 ± 1.66 cm), but the difference did not reach statistical significance (*p* = 0.076). Both groups were predominantly composed of moderately differentiated tumors (56.7% vs. 60.0%), while well-differentiated tumors were numerically more common in MMPCRC than in SMPCRC (22.2% vs. 14.2%), but the overall distribution did not differ significantly between groups (*p* = 0.224).

**Table 2:** Clinical, surgical and pathological features of tumors in patients with MPCRC

Group	SMPCRC (*n* = 120)	MMPCRC (*n* = 45)	*p* value
Tumor location			0.754
Right only	15 (12.5%)	4 (8.9%)	
Left only	42 (35.0%)	15 (33.3%)	
Right and left	63 (52.5%)	26 (57.8%)	
T			0.107
1–2	21 (17.5%)	13 (28.9%)	
3–4	99 (82.5%)	32 (71.1%)	
N			< 0.001*
0	48 (40.0%)	9 (20%)	
1	48 (40.0%)	4 (8.9%)	
2	24 (20.0%)	32 (71.1%)	
Stage			0.188
I-II	48 (40.0%)	13 (28.9%)	
III	72 (60.0%)	32 (71.1%)	
Surgical way			0.035*
Open	3 (2.5%)	5 (11.1%)	
Laparoscopy	117 (97.5%)	40 (88.9%)	
Tumor size (SD), cm	4.29 (1.51)	3.81 (1.66)	0.076
Differentiation			0.224
Low	35 (29.2%)	8 (17.8%)	
Middle	68 (56.7%)	27 (60.0%)	
High	17 (14.2%)	10 (22.2%)	
Total number of lymph nodes harvested (SD)	27.86 (13.32)	20.87 (14.07)	0.004*
Vascular invasion	55 (45.8%)	12 (26.7%)	0.026*
Perineural invasion	50 (41.7%)	14 (31.1%)	0.215
Positive circumferential resection margin	1 (0.8%)	0 (0.0%)	1.000
Mismatch repair status			0.218
dMMR	31 (25.8%)	16 (35.6%)	
pMMR	89 (74.2%)	29 (64.4%)	

Values are presented as counts (percentages), unless stated otherwise

*SMPCRC* Synchronous multiple primary colorectal cancer, *MMPCRC* Metachronous multiple primary colorectal cancer, *SD* Standard deviation, *dMMR* Deficient mismatch repair, *pMMR* Proficient mismatch repair

^*^Statistically significant difference (*p* < 0.05)

**Fig. 2:**
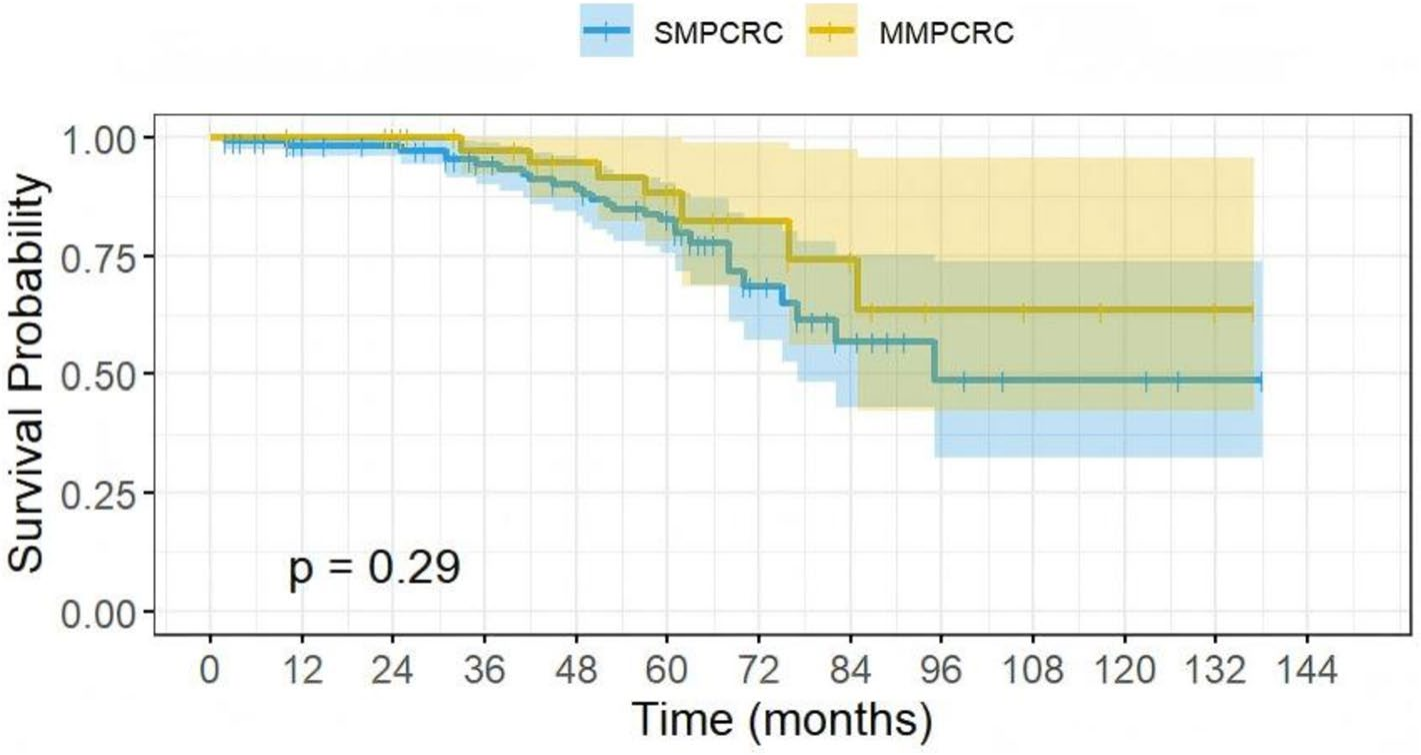
Kaplan–Meier overall survival analysis in patients with multiple primary colorectal cancer. MMPCRC, metachronous multiple primary colorectal cancer; SMPCRC, synchronous multiple primary colorectal cancer

Notably, significantly more lymph nodes were harvested during surgery in the SMPCRC group than in the MMPCRC group (27.86 ± 13.32 vs. 20.87 ± 14.07, *p* = 0.004). Vascular invasion was also more prevalent in SMPCRC (45.8% vs. 26.7%, *p* = 0.026), whereas perineural invasion did not differ significantly between the groups (41.7% vs. 31.1%, *p* = 0.215). Additionally, one case in the SMPCRC group had a positive circumferential resection margin, while none occurred in the MMPCRC group (*p* = 1.000). The prevalence of dMMR tumors was slightly higher in MMPCRC than in SMPCRC (35.6% vs. 25.8%), though this difference was not statistically significant (*p* = 0.218). Survival analysis indicated that there was no significant difference in OS between the two groups (*p* = 0.290, Fig. 2).

### N stage was an independent prognostic factor for the OS in patients with MPCRC

To investigate potential prognostic factors in MPCRC, we performed Cox proportional hazards regression (Table 3). In univariate Cox analysis, demographic factors, personal history variables, tumor location, ASA class, preoperative CA19-9, Hb and ALB levels, surgical approach, mismatch repair status and lymph node yield were not significantly associated with OS. However, elevated CEA levels (HR = 2.602, 95% CI: 0.908–7.456, *p* = 0.075), more advanced T stage (HR = 2.953, 95% CI: 0.894–9.760, *p* = 0.076) and N stage (HR = 3.646, 95% CI: 1.754–7.581, *p* < 0.001), larger tumor size (HR = 2.255, 95% CI: 1.072–4.743, *p* = 0.032), poorer tumor differentiation grade (HR = 1.869, 95% CI: 0.897–3.891, *p* = 0.095), presence of vascular invasion (HR = 2.519, 95% CI: 1.028–6.172, *p* = 0.043) and perineural invasion (HR = 1.897, 95% CI: 0.918–3.919, *p* = 0.084), and positive circumferential resection margin (HR = 6.400, 95% CI: 0.854–47.956, *p* = 0.071) were associated with worse OS, with several showing only a trend toward significance. These eight variables were entered into a multivariable Cox model, which revealed that higher N stage was an independent adverse prognostic factor for survival (HR = 2.968, 95% CI: 1.417–6.287, *p* = 0.005).

**Table 3:** Univariate and multivariate Cox regression analysis for the overall survival of patients with MPCRC

	Univariate Cox regression analysis		Multivariate Cox regression analysis	
Variables (Ref)	Hazard Ratio (95% CI)	*p*-value	Hazard Ratio (95% CI)	*p*-value
Sex (males)	0.822 (0.365–1.852)	0.636		
Age (< 60 years)	1.753 (0.798–3.848)	0.162		
BMI (< 25 kg/m2)	0.897 (0.432–1.861)	0.770		
Smoking history (none)	1.077 (0.512–2.266)	0.845		
Drinking history (none)	1.224 (0.560–2.676)	0.612		
Family history of malignancy (none)	0.891 (0.364–2.181)	0.800		
Previous abdominal surgery (none)	0.981 (0.401–2.402)	0.967		
ASA classification (1–2)	1.593 (0.483–3.547)	0.334		
Preoperative CA19-9 (normal)	1.551 (0.624–3.855)	0.346		
Preoperative CEA (normal)	2.602 (0.908–7.456)	0.075	1.837 (0.872–3.914)	0.109
Preoperative Hb (below mean as Ref)	0.689 (0.326–1.457)	0.330		
Preoperative ALB (below mean as Ref)	0.815 (0.429–1.756)	0.579		
Presence of polyp (none)	1.191 (0.580–2.445)	0.633		
Tumor location (one side only)	1.428 (0.675–3.020)	0.351		
T (T1-2)	2.953 (0.894–9.760)	0.076	1.564 (0.428–5.781)	0.507
N (N0)	3.646 (1.754–7.581)	< 0.001*	2.968 (1.417–6.287)	0.005*
Tumor size (< average)	2.255 (1.072–4.743)	0.032*	1.782 (0.804–4.063)	0.156
Differentiation (low and middle)	1.869 (0.897–3.891)	0.095	1.527 (0.706–3.303)	0.283
Total number of lymph nodes harvested (< 12)	0.536 (0.127–2.268)	0.397		
Vascular invasion (none)	2.519 (1.028–6.172)	0.043*	2.417 (0.934–6.441)	0.069
Perineural invasion (none)	1.897 (0.918–3.919)	0.084	1.193 (0.546–2.608)	0.659
Circumferential resection margin (negative)	6.400 (0.854–47.956)	0.071	2.084 (0.247–17.563)	0.496
Surgical way (open)	0.428 (0.148–1.242)	0.119		
Mismatch repair status (dMMR)	0.912 (0.635–1.260)	0.598		

*Ref* Reference category, defined according to clinical convention or clinical significance

*ALB* Albumin, *ASA* American Society of Anesthesiologists, *BMI* Body mass index, *CA19-9* Carbohydrate antigen 19–9, *CEA* Carcinoembryonic antigen, *CI* Confidence interval, *dMMR* Deficient mismatch repair, *Hb* Hemoglobin, *HR* Hazard ratio, *MPCRC* Multiple primary colorectal cancer, *SD* Standard deviation

^*^Statistically significant difference (*p* < 0.05)

**Fig. 3:**
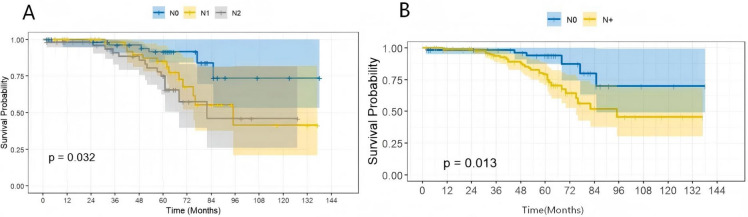
Kaplan–Meier overall survival analysis in patients among different N stages with multiple primary colorectal cancer. **A** N0, N1 and N2 (**B**) N0 and N +

Subsequently, we performed Kaplan–Meier survival analysis with log-rank test to compare the OS of patients of different N stages. The results showed differences in OS across N stages (*p* = 0.032, Fig. 3A). Pairwise comparisons with Bonferroni correction showed a significant difference between N0 and N2 groups (*p* = 0.026), but not between N0 and N1 or between N1 and N2 groups (*p* = 0.202 and *p* = 1.000, respectively, Fig. 3B). Given these results, N1 and N2 stages were combined into a group of positive lymph node metastasis for further survival analysis against the group of negative lymph node metastasis. Patients with node-positive disease had significantly worse OS than those with node-negative disease (*p* = 0.013).

### Expression patterns of MMR proteins in the MPCRC cohort

We then analyzed MMR status (IHC) in all patients. An UpSet plot illustrated the loss patterns of MMR proteins in 47 cases, revealing 14 distinct combinations (Figure S1 in the Supplementary material). The most frequent pattern was combined loss of PMS2 and MLH1, followed by isolated loss of PMS2. Regarding the frequency of protein loss, PMS2 was most commonly lost, followed by MLH1, while MSH6 loss was least observed. An exploratory analysis of MMR status was also performed. No statistically significant difference in overall survival was observed according to MMR status in this cohort. (*p* = 0.620, Figure S2 in the Supplementary material).

## Discussion

In this single-center cohort, synchronous and metachronous MPCRC shared broadly similar baseline profiles, while differences were mainly seen in perioperative management and pathological findings. Patients with synchronous disease more often underwent laparoscopic surgery, had a higher lymph node yield, and showed a higher rate of vascular invasion. However, these subtype-related differences did not translate into a measurable survival separation between the two groups. When the entire MPCRC cohort was analyzed, N stage remained the only independent factor associated with overall survival, highlighting nodal involvement as the dominant determinant of long term outcome in this setting.

Differences between SMPCRC and MMPCRC in surgical approach and lymph node yield are likely influenced by operative context rather than intrinsic biology alone. Synchronous disease is usually treated at initial presentation, when operative planning is more standardized and anatomical planes are less affected by prior dissection, which may facilitate a minimally invasive approach in experienced centers. By contrast, metachronous disease occurs after prior colorectal surgery, and postoperative adhesions and altered anatomy may increase technical complexity and influence operative strategy [13]. Variation in lymph node yield may also reflect differences in surgical strategy, specimen characteristics, and institutional pathology practice [14]. Therefore, these subtype-related perioperative differences should be interpreted primarily in the context of operative complexity and staging evaluation rather than as direct evidence of subtype-specific aggressiveness.

Across the whole MPCRC cohort, nodal involvement carried the strongest prognostic weight. This is clinically relevant because lymph node metastasis reflects regional tumor spread and has direct implications for staging reliability, postoperative risk stratification, and treatment planning. Our finding is consistent with prior MPCRC studies reporting advanced N stage as a key determinant of survival [12, 15]. Although some studies have suggested additional prognostic contributions from factors such as tumor burden or invasion depth, our results indicate that subtype designation alone is less informative than nodal status once patient-level survival is considered [16].

When we examined survival by N stage, the main separation occurred between node-negative and node-positive cases, whereas further stratification within node-positive categories contributed less to discrimination. This pattern supports the clinical view that the presence of regional nodal metastasis marks a meaningful escalation in long-term risk. From a practical standpoint, these findings reinforce the importance of meticulous oncologic resection and adequate nodal evaluation to ensure reliable staging, since insufficient lymph node assessment can lead to stage migration and compromise prognostic stratification [17, 18].

In the present cohort, no statistically significant difference in OS was observed according to MMR status. This finding should be interpreted cautiously. Although dMMR has been associated with favorable prognosis in many localized unifocal CRC cohorts, tumor-based MMR abnormalities do not by themselves establish a diagnosis of LS, and the biological and clinical implications of dMMR in MPCRC may be more heterogeneous [19, 20]. In addition, MMR assessment in this study was based on routine postoperative immunohistochemistry rather than broader molecular characterization, and the limited number of dMMR cases and survival events may have reduced the power to detect modest differences. Therefore, the MMR-related findings of the present study should be regarded as exploratory rather than definitive. Broader molecular characterization will be needed in future studies to better define the clinicopathological and prognostic significance of MMR abnormalities in MPCRC [21, 22].

Several limitations should be acknowledged in this study. Firstly, the retrospective nature of the study carried an inherent risk of selection and information bias. Furthermore, because universal germline testing was not available, occult LS could not be completely excluded among patients with dMMR tumors. Secondly, while the overall cohort size was reasonable, the number of patients with MMPCRC and the final count of survival events might have limited the robustness of the multivariable model and the statistical power for subgroup analyses, as reflected by some wide CIs. Thirdly, the use of a single representative lesion for patient-level analysis may not fully capture the biological heterogeneity across coexisting tumors. Additionally, the conclusions could have been influenced by institution-specific practices in patient management, surgical technique, and pathological assessment in this single-center study, which may limit the generalizability of the findings. Finally, the exploration of molecular characteristics was confined to MMR status, and more comprehensive genomic or transcriptomic profiling might have provided deeper insights into the phenotypic differences observed.

## Conclusion

SMPCRC and MMPCRC differed mainly in perioperative and pathological characteristics, but not in OS. N stage was the only independent predictor of OS in MPCRC, supporting nodal status as the key variable for prognostic stratification. Further multi-center studies with broader molecular characterization are warranted to clarify subtype-specific biology.

## Supplementary Information

Supplementary Material 1.

## Data Availability

The datasets used and analyzed during the current study are available from the corresponding author on reasonable request.
