# Supplementary material for: Nodal stage is the key prognostic factor in synchronous and metachronous multiple primary colorectal adenocarcinoma after curative-intent resection: a retrospective study
Source: World J Surg Oncol. 2026 Apr 28;24:253. doi: 10.1186/s12957-026-04383-7 (PMC13267389; doi:10.1186/s12957-026-04383-7)
Supplement: Supplementary file 1 — Supplementary Material 1. [file 12957_2026_4383_MOESM1_ESM.pdf]

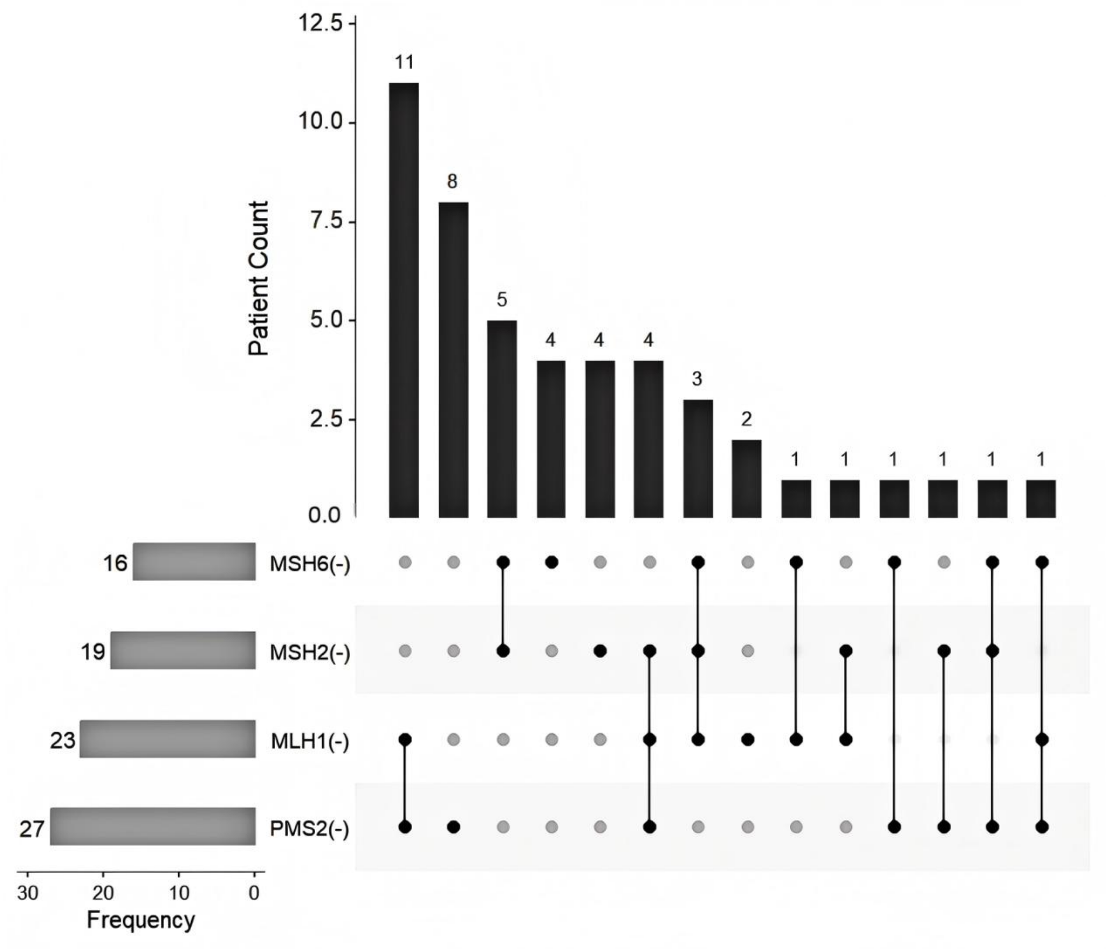

Figure S1 The mismatch repair status in all patients with multiple primary colorectal cancer.

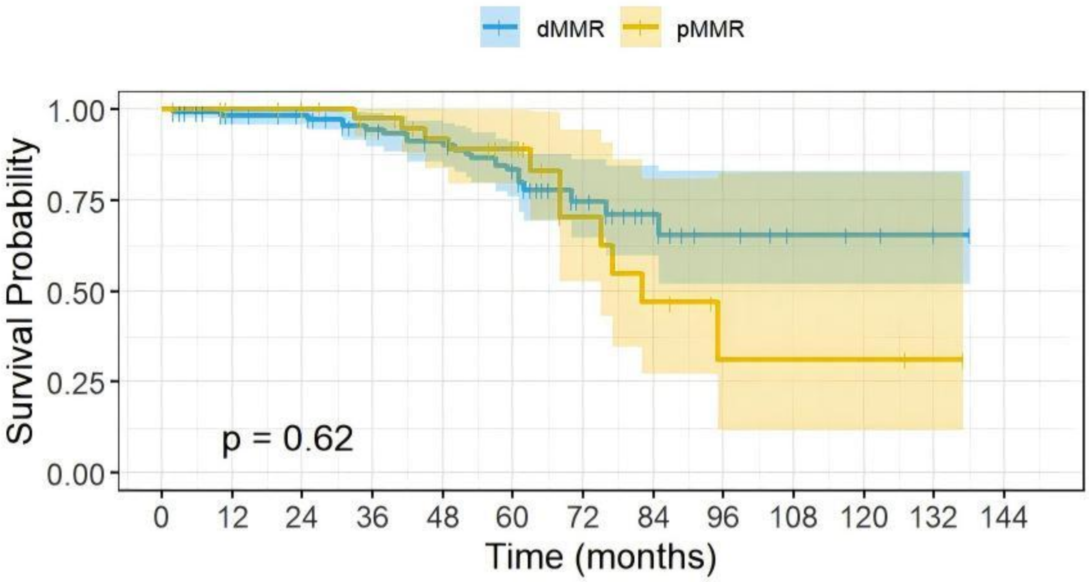

Figure S2 Kaplan-Meier overall survival analysis in patients of different mismatch repair status with multiple primary colorectal cancer. dMMR, deficient mismatch repair; pMMR, proficient mismatch repair.
